# Discovery and Development of Calcium Channel Blockers

**DOI:** 10.3389/fphar.2017.00286

**Published:** 2017-05-29

**Authors:** Théophile Godfraind

**Affiliations:** Pharmacologie, Faculté de Médecine et de Dentisterie, Université Catholique de LouvainBruxelles, Belgium

**Keywords:** calcium channel blockers, voltage operated calcium channels, cardiovascular diseases, hypertension, stroke, dementia, cardiac arrhythmia

## Abstract

In the mid 1960s, experimental work on molecules under screening as coronary dilators allowed the discovery of the mechanism of calcium entry blockade by drugs later named calcium channel blockers. This paper summarizes scientific research on these small molecules interacting directly with L-type voltage-operated calcium channels. It also reports on experimental approaches translated into understanding of their therapeutic actions. The importance of calcium in muscle contraction was discovered by Sidney Ringer who reported this fact in 1883. Interest in the intracellular role of calcium arose 60 years later out of Kamada (Japan) and Heibrunn (USA) experiments in the early 1940s. Studies on pharmacology of calcium function were initiated in the mid 1960s and their therapeutic applications globally occurred in the the 1980s. The first part of this report deals with basic pharmacology in the cardiovascular system particularly in isolated arteries. In the section entitled from calcium antagonists to calcium channel blockers, it is recalled that drugs of a series of diphenylpiperazines screened *in vivo* on coronary bed precontracted by angiotensin were initially named calcium antagonists on the basis of their effect in depolarized arteries contracted by calcium. Studies on arteries contracted by catecholamines showed that the vasorelaxation resulted from blockade of calcium entry. Radiochemical and electrophysiological studies performed with dihydropyridines allowed their cellular targets to be identified with L-type voltage-operated calcium channels. The modulated receptor theory helped the understanding of their variation in affinity dependent on arterial cell membrane potential and promoted the terminology calcium channel blocker (CCB) of which the various chemical families are introduced in the paper. In the section entitled tissue selectivity of CCBs, it is shown that characteristics of the drug, properties of the tissue, and of the stimuli are important factors of their action. The high sensitivity of hypertensive animals is explained by the partial depolarization of their arteries. It is noted that they are arteriolar dilators and that they cannot be simply considered as vasodilators. The second part of this report provides key information about clinical usefulness of CCBs. A section is devoted to the controversy on their safety closed by the Allhat trial (2002). Sections are dedicated to their effect in cardiac ischemia, in cardiac arrhythmias, in atherosclerosis, in hypertension, and its complications. CCBs appear as the most commonly used for the treatment of cardiovascular diseases. As far as hypertension is concerned, globally the prevalence in adults aged 25 years and over was around 40% in 2008. Usefulness of CCBs is discussed on the basis of large clinical trials. At therapeutic dosage, they reduce the elevated blood pressure of hypertensive patients but don't change blood pressure of normotensive subjects, as was observed in animals. Those active on both L- and T-type channels are efficient in nephropathy. Alteration of cognitive function is a complication of hypertension recognized nowadays as eventually leading to dementia. This question is discussed together with the efficacy of CCBs in cognitive pathology. In the section entitled beyond the cardiovascular system, CCBs actions in migraine, neuropathic pain, and subarachnoid hemorrhage are reported. The final conclusions refer to long-term effects discovered in experimental animals that have not yet been clearly reported as being important in human pharmacotherapy.

## Introduction

In 1883, from a series of experiments on isolated heart, Ringer reported that calcium is required for the maintenance of cellular activity. In 1901, Stiles extended this observation to smooth muscle contraction (Ringer, 1883; Stiles, 1901). Sixty years later, Kamada in Japan (Kamada and Kinosita, 1943) and Heilbrunn in the United States (Heilbrunn and Wiercinski, 1947) discovered the role of intracellular calcium for muscle contraction. It is nowadays recognized that calcium is involved in a wide range of cellular processes being generally considered the ubiquitous second messenger.

In the 1960s, experimental work on molecules under screening for coronary dilatation allowed the discovery of the mechanism of calcium entry blockade by drugs later named as calcium channel blockers. Those drugs are now among the most commonly used agents for the treatment of cardiovascular diseases (Abernethy and Schwartz, 1999). The present paper summarizes research on small molecules interacting directly with calcium channels, also considering their therapeutic action effective in cardiovascular diseases and in neurological pathologies and their availability for medical use.

In the early sixties, many drugs have been screened either by imitation or by blockade of a typical effect of identified neurotransmitters. This was before the advent of combinatorial chemistry (Weller, 2000). The chemical structure of the neurotransmitter usually served as initial leading compound for a serial iterative processes of synthesis followed by biological assay. This serial synthesis was a rate-limiting procedure when compared to the current combinatorial chemistry allowing the preparation of many compounds in one time (Swartz, 2000). The bioassay was as simple as possible often avoiding the use of dose effect curves. Structural starting points were natural compounds, dyes or chemical entities already known for other purposes. Usually compounds obtained were termed on the basis of the lead neurotransmitter. For instance, in the group of adrenaline, they were qualified as sympathomimetics or sympatholytics. Nowadays since the deciphering of the genetic code the number of potential targets increased tremendously. In the case of G-protein coupled receptors, there are at least 800 genes representing about 4% of total human genes. Their classification is provided by NC-IUPHAR a Committee of IUPHAR, the international Union of Basic and Clinical Pharmacology.

**Figure 1:**
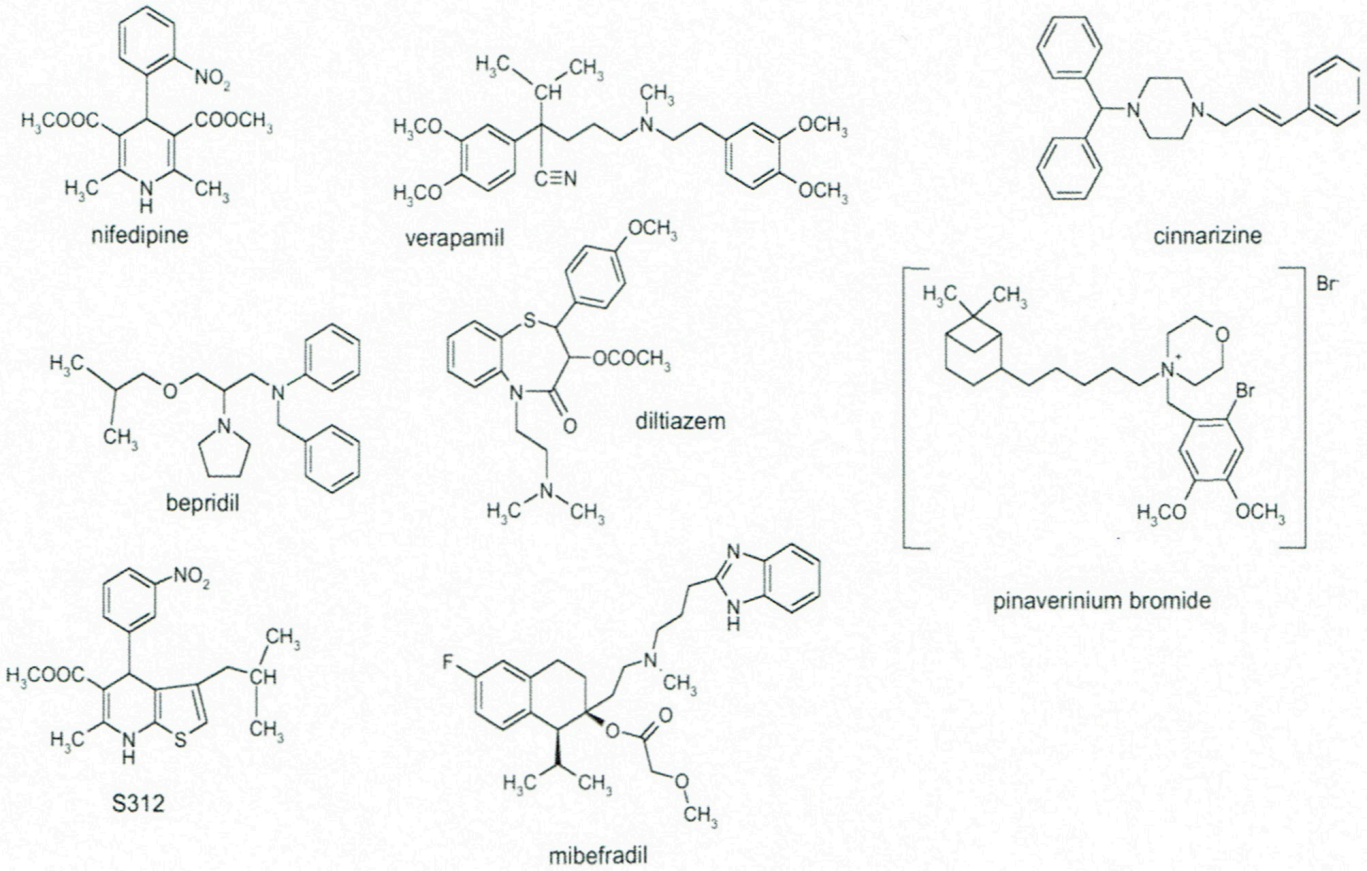
**Chemical structures of the major families of Calcium Channel Blockers as represented by their lead compounds**. Modified from Godfraind (2005).

Coming back to the early sixties, a drug discovery program was started by various pharmaceutical companies targeted coronary circulation in order to discover coronary dilators for the treatment of angina pectoris. This was initiated by Janssen Pharmaceutica with diphenylmethylpiperazines including lidoflazine, cinnarizine, flunarizine (Schaper et al., 1966), and by Knoll AG with phenylalkylamines including verapamil, D600 (Melville et al., 1964). Bayer AG followed with dihydropyridines: nifedipine, nimodipine, nisoldipine (Vater et al., 1972), and Tanabe with the benzothiazepine diltiazem (Sato et al., 1971). Later Sandoz with isradipine (PN200-110) (Hof et al., 1984), Pfizer with amlodipine (Burges et al., 1987), and others followed with other dihydropyridines (Figure 1).

The discovery of calcium channel blockers (CCBs) arose from pharmacological study of screened coronary dilators. This project was included in a program of pharmacological taxonomy initiated in 1964 in Godfraind's laboratory. It aimed to address qualitative and quantitative properties of drugs either under screening or already distributed for medical use. The paper published by Arunlakshana and Schild (1959) was a major basis of this program. It provided essentials of quantitative procedure allowing the definition of the action of antagonists and the estimate of their dissociation constant. Before the publication of this paper, experimental information on antagonist such as type of antagonism, potency, specificity was scarce if not inexistent. However, it must be pointed out that, in 1957, a series of papers on Drug Antagonism had been published in Pharmacological Reviews (vol. 9, Issue 2, 1 Jun 1957). Despite their great theoretical value, none of those papers reached the universal methodological usefulness of Arunlakshana and Schild's publication, which, nowadays, is receiving recognition by the name “The Schild plot” given to the house of the British Pharmacological Society in London.

## From calcium antagonists to calcium channel blockers

**Figure 2:**
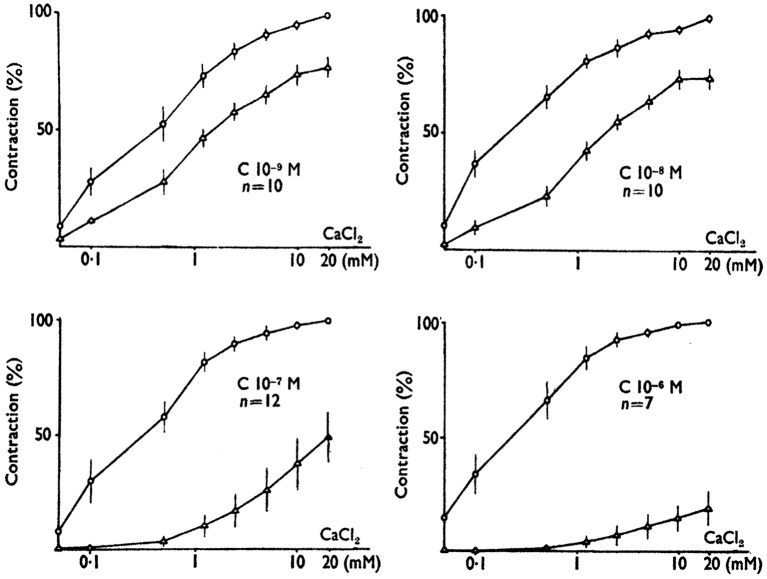
**The effect of cinnarizine on contractions evokes by Ca^2+^ in K^+^-depolarized rabbit mesenteric arteries before and after exposure to various concentrations of cinnarizine**. Responses are expressed as the percentage of maximal contraction evoked before the addition of cinnarizine. Note that the inhibitory effect of cinnarizine is observed at concentration as low as 1 nM and resembles the action of antagonists in receptor studies. This similarity suggested the terminology “calcium antagonist” (Godfraind and Kaba, 1969b; modified).

The first drugs we studied were obtained from Belgian and French Pharmaceutical Companies. In Paul Jannsen's laboratory, Jagenau and Schaper had examined in dog the action of a series of diphenylmethylpiprazines on coronary arteries contracted by angiotensin (Jagenau and Schaper, 1967). Lidoflazine had been selected from this series for clinical studies in patients suffering from angina pectoris (Schaper et al., 1966). The pharmacological action of lidoflazine has been studied by Godfraind et al. on the guinea pig isolated ileum stimulated by angiotensin and other agonists (Godfraind et al., 1966). Collected estimates of pA_2_ and pAh values show similarity of values of pA_2_ and pAh with regard to various agonists studied. Lidoflazine, behaving as an insurmountable antagonist of similar potency for various agonists activating their specific receptors, was hypothesized to act by blocking a mechanism common to those activated receptors. The working hypothesis implied that this common mechanism involved calcium translocation. This hypothesis was based on Edman and Schild's findings that calcium is required in the bathing fluid to obtain a contraction of the Rat uterus in response to acetylcholine (Edman and Schild, 1962). On the basis of the calcium hypothesis, Godfraind and Colleagues designed experiments to examine the activity of depolarized Rat aorta contracted by 10 mM CaCl_2_ and exposed to increasing concentrations of lidoflazine and of other drugs acting similarly. They observed that the calcium-evoked contraction was dose-dependently reduced by lidoflazine, cinnarizine, and chlorpromazine. Furthermore, the antagonist action on Ca^2+^ contraction in various arteries was overcome by increasing Ca^2+^ concentration in the perfusion fluid. On the basis of these observations Godfraind and Colleagues concluded that those drugs were acting as calcium antagonists (Godfraind et al., 1968; Godfraind and Polster, 1968). Further experiments with cinnarizine better determined the nature of this antagonism (Godfraind and Kaba, 1969a). As illustrated in Figure 2, calcium dose-effect curves were performed in depolarized rabbit mesenteric artery in the absence of cinnarizine and 90 min after its addition to the medium. At the lowest concentration of cinnarizine there was a displacement to the right of the calcium dose-effect curve, but at higher concentration, the antagonism was insurmountable. Such observations have been extended to other non-competitive antagonists such as chlorpromazine, papaverine, and several dihydropyridines. The dose-effect curves drawn from these experiments were similar to those obtained in agonist-antagonist studies and it supported the denomination calcium antagonist but it didn't provide indication on the mechanism of this action. Albrecht Fleckenstein and his Colleagues coincidentally made use of the term calcium antagonist in their study on the inhibitory effect of verapamil on electromechanical coupling in mammalian myocardium (Fleckenstein et al., 1969; Spedding and Paoletti, 1992).

In an attempt to better localize the action of calcium antagonists at the cellular level, the contractile response of isolated arteries to catecholamines was further examined in the presence of cinnarizine and of chlorpromazine. In rabbit mesenteric arteries exposed to cinnarizine 10^−6^ and 10^−5^ M, the maximum response to adrenaline recorded in Krebs solution was dose-dependently reduced down to 48 ± 3.9% of the control value with the higher dosage. The maximum response to adrenaline in Ca free solution was equal to 24.1 ± 4.4% of the maximum response in Krebs solution. It was unchanged in the presence of cinnarizine 10^−6^ and 10^−5^ M. Experimental observations were different with chlorpromazine despite its similarity of action on calcium dose-effect curve in depolarized rabbit mesenteric arteries. The maximum response to adrenaline in Krebs solution was dose-dependently depressed by chlorpromazine 10^−7^ and 10^−6^ M down to 26 ± 5.9% of the control value with the higher dosage. Responses to adrenaline in Ca free solutions were dose-dependently lowered with chlorpromazine 10^−7^ and 10^−6^ M down to 4.5 ± 1.1% with the higher dosage. Since by contrast with chlorpromazine, cinnarizine didn't alter the contraction evoked by adrenaline in calcium-free solution, it appeared unlikely that it acted as an antagonist of calcium on an intracellular target. Extracellular calcium being necessary to reach full artery contraction, a rational working hypothesis was that cinnarizine-dependent blockade of calcium entry occurred specifically at the level of cellular membrane, which was not the case for chlorpromazine that likely displayed an intracellular effect on contraction in addition to its action on calcium entry (Godfraind and Kaba, 1969a).

**Figure 3:**
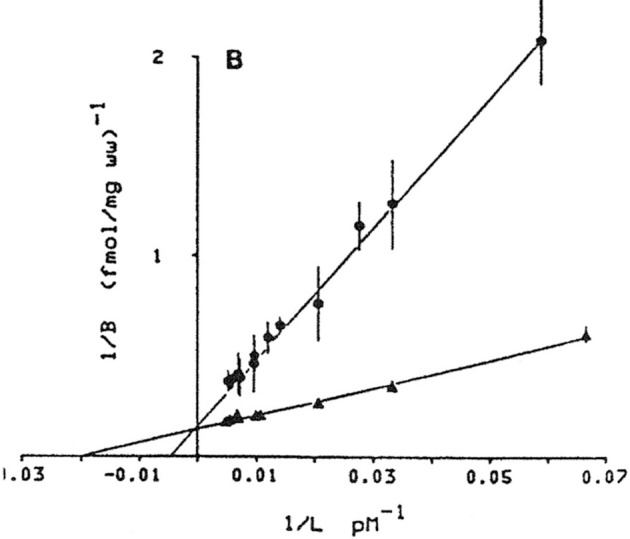
**Influence of membrane potential on isradipine binding affinity in intact artery exposed (▴) or not (•) to K^+^ depolarization**. Double-reciprocal plot showing change in affinity but no change of Bmax (Morel and Godfraind, 1987, 1991).

Studies of Ca^2+^ exchange in intact arteries have tested this hypothesis trough a comparison of dose-effect curves for inhibition of contraction and calcium fluxes in the absence and presence of calcium entry blockers. Calcium fluxes we measured with La^3+^, which has roughly the same hydrated radius than Ca^2+^ but due to higher valence has a higher affinity than calcium for calcium binding sites. Furthermore, lanthanum doesn't enter the cell. The rate of change of the ^45^Ca content of the tissue washed in lanthanum solution provides an estimate of the Ca fluxes across the smooth muscle cell membrane. Lanthanum was used for this purpose in various smooth muscles by several authors (Weiss and Goodman, 1969; Van Breemen et al., 1972; Karaki and Weiss, 1979). In isolated rat aorta under activation of adrenoceptors, Godfraind observed (Godfraind, 1976) a dose-dependent increase of the rate of ^45^Ca uptake. Furthermore, in vessels loaded with ^45^Ca solution, the rate of ^45^Ca loss in normal solution is increased by noradrenaline. Phentolamine displaced to the right the dose effect curves of the action of noradrenaline on ^45^Ca uptake, an effect typical of competitive antagonism with a pA_2_ of 7.8, a value close to that found in contraction studies. This indicates that the activation of α-adrenoceptors is responsible for both Ca entry and contraction. Godfraind and Dieu showed that flunarizine blocks both norepinephrine- and depolarization-dependent ^45^Ca influx while ^45^Ca efflux is not significantly modified (Godfraind and Dieu, 1981). The action of nifedipine is similar, but on a quantitative basis, nifedipine is more active as blocker of KCl than of norepinephrine-evoked effects. This is substantiated by IC_50_value equal to 1.7 × 10^−8^ M with norepinephrine and 1.6 × 10^−9^ M with KCl-depolarization. Concentration inhibitory curves for Ca influx and contraction are superimposed suggesting that action on contractility is related to blockade of calcium entry through channels opened during adrenoceptors activation (Godfraind, 1983). As shown by Morel and Godfraind (1987), in the presence of dihydropyridines, the KCl-dependent contraction is reduced as a function of concentration of the blocker and of duration of the depolarization, indicating that membrane potential has an effect on dihydropyridine action. Specific binding of dihydropyridines also depends on the duration of depolarization, signifying that specific binding sites on calcium channels undergo a membrane potential dependent modification as illustrated in Figure 3 (Morel and Godfraind, 1987). There are three main categories of calcium channels: receptor activated channels (RAC), ligand-gated channels (LGC), and voltage-operated (VOC). Briefly, VOCs are transmembrane hetero-oligomeric complexes (Catterall, 2011). A report on nomenclature (Ertel et al., 2000) and classification (Catterall et al., 2005) of voltage-operated calcium channels is provided in the IUPHAR/BPS Guide to Pharmacology accessible at http://www.guidetopharmacology.org. Calcium channel main component is the α1 subunit, which is pore-forming containing binding sites for CCBs. The 10 cloned α1 subunits are grouped into three families (Table 1).

**Table 1:** **Nomenclature of voltage-operated calcium Channels (Ertel et al., 2000; Catterall et al., 2002)**.

**Type**	**a_1_-subunit**	**Splice**	**Current**
Ca_v_ 1.1	α_1_ 1.1		L
Ca_v_ 1.2	α_1_ 1.2	Ca_v_ 1. 2a	L
		Ca_v_ 1.2b	L
		Ca_v_ 1.2c	L
Ca_v_ 1.3	α_1_ 1.3		L
Ca_v_ 1.4	α_1_ 1.4		L
Ca_v_ 2.1	α_1_ 2.1	Ca_v_ 2.1a	P/Q
		Ca_v_ 2.1b	
Ca_v_ 2.2	α_1_ 2.2	Ca_v_ 2.2a	N
		Ca_v_ 2.2b	N
Ca_v_ 2.3	α_1_ 2.3	Ca_v_ 2.3a	R
		Ca_v_ 2.3b	R
Ca_v_ 3.1	α_1_ 3.1		T
Ca_v_ 3.2	α_1_ 3.2		T
Ca_v_ 3.3	α_1_ 3.3		T

The structure of those channels was initially published for channels extracted from striated muscle (Numa et al., 1990). Each α1 subunit has four repeats, each with six transmembrane domains and the pore-forming region between the two transmembrane domains S5 and S6. When inserted in lipid bilayer it exhibits properties of calcium channel, including receptor binding sites for the various CCBs. A recent structural study of bacterial Ca_V_ channel confirmed the different locations of the binding sites of amlodipine and verapamil. Crystallographic analysis showed that amlodipine and other dihydropyridines block the channel pore by interacting with its external, lipid-facing surface but that verapamil interacts with the intracellular side of the selectivity filter and blocks the ion-conducting pathway located in the central cavity of the pore (Tang et al., 2016). Calcium channels found in heart and vessels (L-type) are splicing products of a same gene. Isoforms are distributed unequally among the various cardiac and smooth muscles (Feron et al., 1994). Lowercase letters are used to distinguish alternatively spliced variants; Ca_V_1.2a corresponds to channels containing the cardiac variant and Ca_v_1.2b corresponds to the smooth muscle variant (Welling et al., 1997). Thus, Cav1.2 (L-type) channels are the channels operating in cardiac and smooth muscles. They are high-voltage activated and blocked by CCBs. The modulated receptor model may help to describe the interaction of CCBs with voltage-operated calcium channels. The modulated receptor theory was initially developed by Hille (1977) for local anesthetic actions on sodium channels (Godfraind, 1986). The theory proposes that the state of the channel influences the drug binding to a site located within the channel and that this state is determined by membrane potential. It has been extended to calcium channels in cardiac muscle in which blockade of calcium current by dihydropyridines calcium entry blockers is modulated by membrane potential (Lee and Tsien, 1983). Block is more pronounced when calcium current is measured during voltage clamp pulses applied from depolarizing holding potentials. In preparations that are not voltage clamped, blocking activity is influenced by cell resting potential (Sanguinetti and Kass, 1984). The model proposed by Bean for cardiac cell provides analysis of the voltage-dependence of binding of isradipine to intact arteries (Bean, 1984). As already mentioned above, K-evoked depolarization changes the affinity of CCB dihydropyridines. According to the model, the channel might present three convertible states: the resting state predominating in polarized cells where the channel is closed but available for opening, the open or activated state that is promoted by depolarization pulses beyond a certain threshold, the inactivated state unavailable for opening that is favored by prolonged depolarization. Most 1,4-dihydropyridines bind preferentially to the inactivated state of Cav1.2 channels. Therefore, depolarizing holding potentials that increase the proportion of inactivated channels, but fail to open them, enhance inhibitory potency because drug binding to inactivated channels decreases the proportion of channels that are available for activation. Assuming that K_L_ and K_H_ are dissociation constants for respectively low-affinity and high-affinity states and that, in the absence of the drug, L is the fraction of channels in the resting state, the model predicts that at any given holding potential, the concentration dependence of drug binding, and thus of calcium current inhibition, follows a simple adsorption isotherm with an apparent dissociation constant that is given by:

$$\begin{array}{l} {\text{K}}_{\text{app}}=1/\left[\left(\text{L}/{\text{K}}_{\text{L}}\right)+\left(1-\text{L}\right)/{\text{K}}_{\text{H}}\right] \end{array}$$

Experimental data in various organs validated this model (Bean et al., 1986; Yatani et al., 1987; Nelson and Worley, 1989; Godfraind et al., 1992a)

Such experiments allowed the pharmacological identification of CCBs with a higher affinity for inactivated than for closed channels. Those may be termed voltage-dependent CCBs. They belong to the group characterized by a high vasoselectivity. The degree of initial inhibition of a vasoconstrictor stimulus may differ along the vascular tree since the resting membrane potential varies between vessels, the apparent affinity of a voltage-dependent CCBs for Ca^2+^ channels being related to the resting membrane potential (Cauvin and van Breemen, 1985). Cerebral microvessels, which display a resting membrane potential much lower than peripheral vessels, are more sensitive to the voltage-dependent CCB nimodipine than conduit arteries. This accounts for the selectivity of nimodipine in the cerebral circulation (Morel and Godfraind, 1989). The role of voltage-dependent binding on vascular selectivity has also been observed by Sun and Triggle in a large series of dihydropyridines (Sun and Triggle, 1995).

## Tissue selectivity of calcium channel blockers

From the previous section, it is obvious that the blocking action of CCBs on induced contraction of vessels is depending on the resting membrane potential, this action is considered selective for a given condition. Indeed the term “tissue selectivity” is used when an agent shows different degrees of potency between tissues and a preferential action in a given one. This is well-known for adrenoceptors when considering the differences between α-adrenoceptors and β-adrenoceptors for both agonists and antagonists. Several factors may be involved in tissue selectivity when considering characteristics of a given drug, properties of a given tissue and characteristics of the acting stimulus. Some examples need to be given (Godfraind et al., 1986a,b).

### Characteristics of the drug

Electrophysiological studies of various compounds considered their relative potency on L- and T-type currents. In a study with verapamil, diltiazem, lacidipine, and minefradil, it was observed that all blocked both T and L channels, but that their selectivity was differing. In this selection, mibefradil is the most selective for T-channels and lacidipine for L-channels (De Paoli et al., 2002). As shown by Furukawa et al. (1997) amlodipine has a strong blocking action on both L- and N-type calcium channels expressed in oocytes. The level of the amlodipine block on the N-type Ca^2+^ channel is similar to that on the L-type Ca^2+^ channel. The concentration dose-effect curves are nearly superimposed. IC50 values for amlodipine block on the L-type and N-type Ca^2+^ channel are 2.4 and 5.8 μM respectively at −100 mV holding potential. Action of amlodipine on the N-type Ca^2+^ channel is dependent on holding potential and extracellular pH, as observed with amlodipine block on L-type Ca^2+^ channel. Blocking action of amlodipine is enhanced by depolarized holding potential and high pH. Time course of block development by amlodipine is similar for L-type and N-type Ca^2+^ channels, but slower than the time course of block development by nifedipine for L-type Ca^2+^ channel. Amlodipine is also active on T-type currents. Bénardeau and Ertel (1998) have reported an IC_50_ value of 5.6 μM for T- channel block in guinea-pig atria. It is of note that in this concentration range, amlodipine is a powerful ACE inhibitor, which through the preservation of bradykinine stimulates the release of NO from endothelial cells (Xu et al., 2002). This latter effect is important in view of the synergism for vascular relaxation existing between NO and CCBs (Godfraind and Salomone, 1996; Salomone et al., 1996). Those various properties of amlodipine need to be taken into account when examining the therapeutic mode of action of this drug. Edward Perez-Reyes et al have studied several CCBs on recombinant Ca_v_3.2 channels (Perez-Reyes et al., 2009). They noted that four clinically approved antihypertensive drugs (efonidipine, felodipine, isradipine, and nitrendipine) are potent T-channel blockers (IC_(50)_ < 3 microM). However, highly prescribed dihydropyridines, such as amlodipine and nifedipine, are 10-fold less potent on T-channels than on L-channels, these are more appropriate for use in research studies on blockade of L-type currents in therapy. Cilnidipine is highly potent against N-type current (Uneyama et al., 1997). It may be anticipated that those CCBs acting at the level of N-type currents and thereby impairing catecholamines release from nerve endings, should blunt the sympathetic reflex following vasodilatation. This is the basis of another difference of selectivity between drugs. Therefore, it is appropriate to consider the selectivity window, which can be related not only to the ratio of active concentrations blocking L-type channels and other channels (T or N-type) but also to the ratio of active concentrations blocking L-type channels and other membrane processes (such as receptors, other channels, or transporters). For instance D600 blocks not only L-type channels but also the α-adrenoceptors (Godfraind et al., 1992b). Dihydropyridines may also block Na channels, but at higher concentration than calcium channels (Yatani et al., 1988). Another example is flunarizine, which does not only interfere with the various voltage-operated calcium channels, but which is also acting on the release of dopamine by nerve terminals (Terland and Flatmark, 1999). Great attention has been devoted to the selectivity for other ion channels, in particular K-channels, verapamil has the lowest ratio CaCh/KCh of the drugs tested. It is acting at nearly the same concentration on both channels; the consequences of this property have not yet been evaluated (Grace and Camm, 2000; Hatano et al., 2003). Interestingly, the two splice types of α_1_ subunit have different sensitivity for nisoldipine a dihydropyridine (DHP) Ca^2+^ channel blocker with a high vascular selectivity (Godfraind et al., 1992b). Nisoldipine is a more powerful blocker of inward current in cells transfected with α_1_1.2b isoform cDNA than in those transfected with α_1_1.2a isoform cDNA. Nicole Morel et al have examined if this property is shared by other DHP and non-DHP Ca^2+^ channel blockers (Morel et al., 1998). They used Chinese hamster ovary cells (CHO) transfected either with cDNA encoding for the α_1_1.2a or with cDNA encoding for the α_1_1.2b subunit of the L-type Ca^2+^ channel, issuing respectively from rabbit heart and lung smooth muscle (Welling et al., 1993). The Ca^2+^ channel blocking activity of three neutral DHP derivatives, (+)-PN 200-110, nifedipine and nisoldipine, which show different degrees of vascular selectivity (Godfraind et al., 1992a,b), and one positively charged derivative SDZ 207-180 (Kass et al., 1991) was compared to that of the phenylalkylamine verapamil, which is equipotent in cardiac and vascular tissue (see below) and to that of pinaverium bromide, a non-DHP compound with a quaternary ammonium, reported to show intestinal selectivity (Christen, 1990). The voltage-dependent current mediated by the α1 subunit of the L-type Ca^2+^ channel (Iα_1_) was recorded with the whole-cell configuration of the patch-clamp technique using barium ions as charge carrier. Binding affinity of Ca^2+^ channel blockers was also assessed in displacement studies using the Ca^2+^ channel ligand [^3^H]-(+)-PN 200-110. Experimental results show that neutral dihydropyridines (nifedipine, nisoldipine, (+)-PN200-110) were more potent inhibitors of α_1_1.2b subunit than of α_1_1.2a subunit. This difference was more marked at a holding potential of −100 mV than at −50 mV. SDZ 207-180 (an ionized dihydropyridine) exhibited the same potency on the two isoforms. Pinaverium (ionized non-dihydropyridine derivative) was 2-and 4-fold more potent on α_1_1.2a than on α_1_1.2b subunit at Vh of −100 mV and −50 mV, respectively. At both voltages, the two isoforms were equally sensitive to verapamil. Neutral dihydropyridines had a higher affinity for the α_1_1.2b than for the α_1_1.2a subunit as shown by binding experiments with [^3^H]-(+)-PN 200-110. SDZ 207-180 had the same affnity for the two isoforms and pinaverium had a higher affinity for the α_1_1.2a subunit than for the α_1_1.2b subunit.

All these results show that marked differences are observable among Ca^2+^ channel blockers concerning their selectivity not only for the three subfamilies of calcium channels but also for the α_1_1.2a and α_1_1.2b subunits. Therefore, this justifies studies on the pharmacological profile of drugs belonging to a same chemical family in order to see if they present or not uniformity in action.

### Properties of the tissue and characteristics of the stimuli

In order to characterize these factors, we may focus on some characteristics of the selectivity of CCBs in the cardiovascular system. They were identified in experiments with arteries and myocardium isolated in different species. In view of species-dependent selectivity, data obtained with human preparations are important for translational Medicine. Indeed, potency ratios of nifedipine expressed as ratio of IC_50_ for cardiac inotropism over IC_50_ for contraction of depolarized vessel are dissimilar within species, the highest value being obtained in rat and the lowest in guinea pig. Comparing various CCBs in guinea-pig, Spedding et al. (1990) showed that the ratio of IC50 values heart/vessels is 0.3 for diltiazem, 1.3 for verapamil and 3.1 for nifedipine. Experiments from Triggle's laboratory are in complete agreement with the results just mentioned (Triggle, 1999).

**Figure 4:**
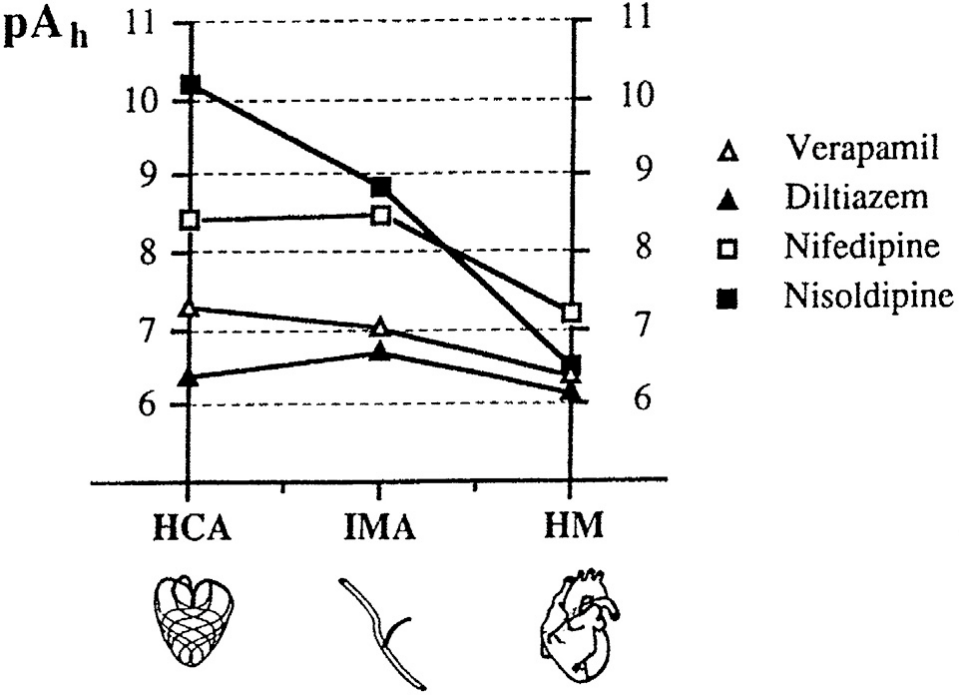
**Logarithmic scale of the IC_50_ (pA_h_) of CCBs in human coronary (HCA) and internal mammary (IMA) arteries stimulated by serotonin and in electrically stimulated human myocardium (HM)**. IC_50_ is the concentration producing 50% reduction of the contraction. Modified from Godfraind et al. (1992b).

^3^H(+)-isradipine, was used in order to estimate the apparent affinity of dihydropyridine-binding sites in plasma membranes of human coronary artery and human myocardium. The sequence of affinity was nisoldipine > isradipine > nifedipine. The dissociation constant values may be compared to functional values of IC_50_ obtained in other experiments (Godfraind et al., 1992b). Functional estimates of IC_50_ of CCBs in human coronary arteries exposed to serotonin are close to radioligand estimates of the apparent dissociation constant in corresponding plasma membranes. In human heart, functional IC_50_ values are much higher than radiochemical K_i_ (or K_d_) values estimated in plasma membranes. There is a 10,000-fold difference for nisoldipine, a 1,298-fold difference for isradipine and a 27-fold difference for nifedipine. Nisoldipine is much less active than nifedipine in cardiac preparation and much more active in human coronary artery. Differences between CCBs are illustrated in Figure 4, which illustrates log scale of the reciprocal of the concentration needed to block 50% of the contractile activity (IC_50_) in human coronary artery, human internal mammary artery, and human myocardium. It shows that diltiazem and verapamil have the same potency in arteries and in myocardium. For nisoldipine, the potency sequence is coronary artery >> mammary artery >> myocardium, whereas for nifedipine, the sequence is coronary artery = mammary artery >>myocardium (Godfraind et al., 1992b). Other experiments (Sarsero et al., 1998), using preparations from human atria and aortic vasa vasorum, confirm the vasoselectivity of nisoldipine, and the cardioselectivity of verapamil. Several factors might be involved in the difference in sensitivity between heart and vessels. In cardiac myocytes, sarcolemmal Ca channels bring Ca into the cell (L- and T-type Ca channels). This Ca influx contributes an inward current, which makes (or keeps) the membrane potential more positive and activates contraction, being second messenger in the excitation-contraction (E-C) coupling. It activates intracellular Ca channels allowing the release of Ca from the sarcoplasmic reticulum (SR) and endoplasmic reticulum (ER; ryanodine and IP_3_ receptors), which amplifies the function of Ca that enters via the sarcolemma (Bers and Perez-Reyes, 1999). The ryanodine receptors (RyR) are more numerous that the L-type channels in the sarcolemma. According to Wibo et al. (1991), the ratio is 1 over 9.

**Figure 5:**
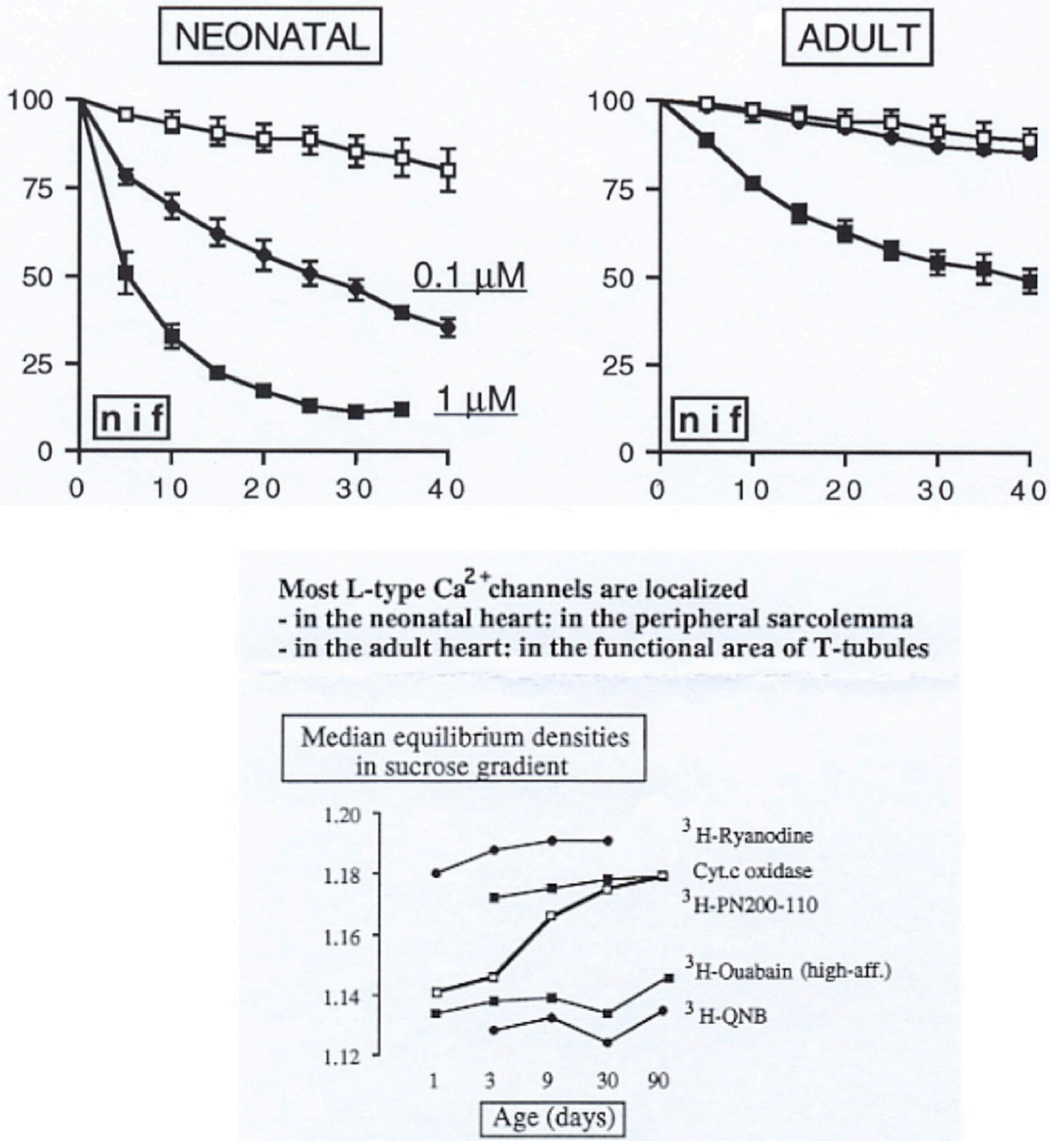
**Upper graphs show different sensitivity to negative inotropic action of nifedipine in neonatal and adult heart**. Lower graph shows cellular location change of CCB as a function of age in rat heart. Modified from Wibo et al. (1991).

The cardiac E-C mechanism is influenced in different circumstances. These include autonomic modulation, L-arginine-NO pathway activation and pathological conditions related or not to hypertension and atherosclerosis (Balligand et al., 1993; Maier and Bers, 2002). The classical long plateau of cardiac action potentials is essential for preventing re-excitation and arrhythmias. As already pointed out above, the α_1_-subunit is one among the five subunits constitutive of voltage-operated calcium channels. When inserted in lipid bilayers, it shows properties of calcium channels particularly the binding sites for the various CCBs. Different genes are coding the various calcium channels types. Nevertheless, splicing products of the same gene such as l-type calcium channels found in heart and vessels have different affinities for dihydropyridines, but not for verapamil. This observation is consistent with the absence of vascular selectivity of verapamil (see above). However, differences in sensitivities between heart and vessels observed in pharmacological experiments don't appear to be only due to affinity ratio and other factors need to be considered (Morel et al., 1998). Feron et al. (1994) have reported tissue-dependent developmental regulation. During development, there is a profound modification of the localization of L-type Ca channels from the peripheral plasma membrane to the junctional structures (Figure 5) (Wibo et al., 1991). The functional consequence results in a lower sensitivity to CCBs of adult over neonatal hearts (Wibo et al., 1991).

Time-course experiments may provide additional information on the tissue selectivity. The inhibitory action of nisoldipine and other dihydropyridines on contractions is characterized by a marked time-dependency following initiation of the depolarizing stimulus in pre-incubated arteries: the inhibition increases slowly after depolarization to attain a steady-state value. By contrast, in the presence of verapamil and diltiazem, the inhibitory action is immediate, and sustained for the duration of the depolarization. Inhibition by dihydropyridines follows the kinetics of their binding to receptors connected to different modulations of calcium channels due to variable durations of the stimuli. The rate of association in intact depolarized tissues follows a pseudo-first-order kinetics similar to the association rate constant for purified calcium channels (Wibo et al., 1988). The short systolic depolarization (±0.4 s) does not favor a proportion of inactivated calcium channels similar to that obtained after the longer stimulus duration (±6 min) required to full activation of vascular smooth muscle. This controls the concentration of nisoldipine required to occupy a given proportion of inactivated channels for a short systolic depolarization or for a time required to fully activate a smooth muscle. For an occupation of 50 per cent of receptor sites at the end of the stimulus, the ratio of concentrations in cardiac vs. arterial muscle is equal to 1,500, a value similar to that found in contraction studies (Godfraind et al., 1987). *In vivo* studies are fully consistent with *in vitro* studies (Rousseau et al., 1994).

The degree of inhibition of response to a given vasoconstrictor may be different between arteries exposed to the same CCB. The curves relating inhibition by nisoldipine of serotonin-evoked tonic contraction in various arteries are not superimposed. For instance inhibition of the tonic contraction to serotonin is greater in human coronary artery than in human internal mammary artery. This extends earlier reports on inhibition of contractile responses depending on the type of vessel: resistance arteries being more inhibited than conduit arteries (Godfraind and Polster, 1968; Godfraind et al., 1968). Another illustration of heterogeneity is related to the mode of activation of the contraction by various adrenoceptor agonists. The maximum contractions of rat aorta evoked by α_1_-agonists noradrenaline or phenylephrine are less inhibited than maximum contractions evoked by α_2_ agonists such as clonidine and oxymethazoline. Such observations have been extended to various CCBs and to various vessels. For instance, the maximal contraction evoked by ET-1 in human isolated coronary arteries exposed or not to nisoldipine (1 μM) (Balligand and Godfraind, 1994) is inhibited by 51% in distal arteries whereas it is inhibited by only 35% in proximal segments. They emphasize the hypothesis that vascular heterogeneity may be, at least partly, related to the proportion of contractile responses resistant or not to calcium-channel blockade (Godfraind, 1994). It is likely that the interaction of agonists with their receptors activates targets other than voltage-operated calcium channels such as protein kinase C, thus resulting in an increase in the contractile proteins sensitivity to calcium (Karaki, 1989; Nishimura et al., 1990). The importance of this mechanism that is independent of Ca entry is likely to vary between different smooth muscles and to play a role in vascular selectivity. Among factors responsible for heterogeneity among vessels, not only the specificity of the blocker and its voltage-dependency but also its tissue pharmacokinetics needs to be taken into account. For instance, Angelico et al. (1999) have observed in isolated rabbit aorta stimulated by high KCl that 50% relaxation was reached at different times according to the CCB tested. At 10 nM concentration 50% relaxation was reached after 210 min with lercanidipine, 278 min with amlodipine, 135 min with lacidipine, 75 min with nitrendipine, and 70 min with felodipine. On the other hand, when studying the heart ventricle, the rate sequence was lacidipine > amlodipine > felodipine > lercanidipine = nitrendipine, indicating that comparison between drugs requires appropriate experimental conditions in order to validate the IC_50_ ratio. It is noticeable that arteries and veins display different sensitivities to CCBs and to various vasoconstrictors. This has been documented with endothelin (ET1) in rings of canine vessels (Miller et al., 1989) and in isolated human vessels. In the latter, the contractile response of coronary artery to ET1 is blocked by CCBs by contrast the response of coronary vein is insensitive (Balligand and Godfraind, 1994). This differential sensitivity is also observable with noradrenaline (Sjoberg et al., 1987) as well as on arteries and veins in humans (Robinson et al., 1980).

## Acute hemodynamic effects of CCBs

### Systemic hemodynamic

Calcium channel blockers are used in therapy for long-term even life-long-treatment, nevertheless it is of interest to examine the acute hemodynamic changes evoked when those agents are administered at pharmacologically active doses. Such a study allows comparing the various CCBs from a functional point of view considering potency, pharmacokinetics and tissue selectivity. The hemodynamic action of CCBs has been studied *in vivo* with most of the compounds, once they were identified *in vitro*. Due to the complexity of the CCBs-evoked hemodynamic reflexes and to their tissue selectivity, those drugs exhibit variations in action according to the vascular bed or the species so far considered particularly at the level of coronary, renal, and cerebral circulations. In conscious rats, Ishii et al. (1980) have observed that nifedipine decreased blood pressure at doses much lower in SHR than in WKY. Knorr and Garthoff (1984) compared nitrendipine and hydralazine in SHR and WKY. They reported that the vasodilator hydralazine is equipotent in both strains at variance with nitrendipine less active on blood pressure in WKY than in SHR. Consistent with hemodynamic observations (Kazda and Knorr, 1990), these studies show that the activity profile of CCBs in cardiovascular system is different from the activity profile of classical arteriolar vasodilators. Furthermore, during chronic administration, the decrease of blood pressure occurs without change of the cardiac frequency (Frohlich, 1986).

Following the administration of increasing intravenous doses of nitrendipine. Taylor et al. (1984) there is a progressive reduction of blood pressure, reaching a maximum effect at 50% of initial value, accompanied by a reduced resistance and an increase of blood flow in the femoral and mesenteric territories. Taylor and Colleagues showed that CCBs are arteriolar dilatators with differing activities according to the circulation bed with greatest effect in the coronary bed. It is important to note that no significant hemodynamic effects have been observed in the venous circulation. In dog the coronary circulation is more influenced that the peripheral one. Acute response to IV administration evokes major sympathetic reflexes characterized by an increased cardiac rhythm. In human after sublingual nifedipine, the vasodilator action does occur mainly on the arterial side with a modest effect on the venous side (Merillon et al., 1980). It is worth mentioning that ACE inhibitors dilate to the same extent the arterial and the venous beds. Variations in regional sensitivity to the vasodilatation effects of CCBs are in agreement with *in vitro* vascular selectivity. Following the injection of nifedipine there is a large increase in coronary flow with reduced myocardial oxygen consumption. Dilatation of resistance vessels in the coronary bed overcomes reflex coronary vasoconstriction occurring physiologically when myocardial oxygen consumption is decreasing (Berdeaux et al., 1990).

### Mesenteric and renal beds

Janssen et al. (2001) have studied in rats the regional hemodynamic effects of long acting CCBs barnidipine and amlodipine. In male adult SHRs, Doppler flow probes and catheters allowed the measure of renal (RVR), mesenteric (MVR), and hindquarter (HQVR) vascular resistance changes. One week after surgery, barnidipine, or amlodipine were intravenously administered at three doses causing comparable reductions in mean arterial pressure (MAP). Barnidipine at doses of 3, 10, and 30 μg/kg reduced MAP (± SEM) by 8 ± 2, 26 ± 3, and 45 ± 4 mmHg (*n* = 10). Amlodipine achieved similar effects on MAP at doses of 100, 300, and 1,000 μg/kg. Barnidipine at 3 and 10 μg/kg reduced MVR (% ± SEM) by 4 ± 4 and 19 ± 4, and RVR by 8 ± 2 and 15 ± 4, respectively. In contrast, HQVR remained unaltered. Similar observations were done with amlodipine, except that changes in RVR were half of those found after barnidipine. By contrast short-acting nifedipine and isradipine reduced HQVR and not RVR.

### Cerebral blood flow

Change in blood flow resulting from increase in cardiac output has consequences in tissue perfusion according to the vascular bed. Physical exercise producing a 3-fold increase in cardiac output leads to a 10-fold increase in blood flow to skeletal muscle, halves renal blood flow, but does not modify cerebral blood flow (Wade and Bishop, 1962). Antihypertensive drugs can have pronounced effects on CBF. As discussed by Atkinson and Capdeville (1990), those agents can be divided into three categories. In the first are found those that decrease CBF, which can further be subdivided into drugs which interfere with noradrenergic vascular tone such as ganglion blocking agents, centrally acting hypertensive drugs like clonidine, alpha adrenoreceptor blocking agents such as prazosin and various vasodilators such as diazoxide, sodium nitroprusside and nitroglycerin. A second group is composed of drugs that have minor effects on CBF. They include antagonists of the vascular 5-HT2 receptor such as ketanserin, that markedly increase cardiac output but produce very little change in CBF, blood flow being distributed to the gut, kidneys and skeletal muscles. Likewise beta-blockers and ACEI have no significant effect on CBF. A third category of antihypertensive drugs is composed of those that increase CBF, including CCBs. The increase in cardiac output produced by CCBs, such as felodipine, for example, is accompanied by a marked increase in CBF, similar to that observed in coronary blood flow, and far above that seen in other organs such as the kidney, the gut, and the skeletal muscles (Bolt and Saxena, 1984). This pharmacological profile is shared with other vasodilators such as potassium channel activators and hydralazine, but the cerebrovascular dilator effect of dihydropyridine CCBs is the most pronounced.

Hara et al. (1999) have studied in anesthetized rats the effects of lomerizine, a CCB of the piperazine group, on cerebral blood flow by laser Doppler flowmetry and on vertebral blood flow in anesthetized beagle dogs with an electromagnetic flowmeter. They observed that lomerizine (2.5 and 5 mg/kg, intraduodenally) dose-dependently increased vertebral blood flow in dogs without significantly changing BP or HR. With 10-mg/kg intraduodenal lomerazine, vertebral blood flow remained elevated from 20 to 240 min after administration despite a concomitant decrease of BP occurring from 20 to 120 min. This confirms the concept reported above that CCBs might oppose the vasoconstrictive reflex in cerebral vessels, which is physiologically activated in response to a fall in blood pressure.

### Regional response and differences between CCBs

Studies on anesthetized dogs help to characterize differences between molecules, taking into account their potency, the time course of their effect and their specificity of action on a given vascular bed. In view of renal hemodynamic, actions on renal blood flow, and on natriuresis provide the opportunity to clarify the mechanisms involved in the therapy of hypertension. Calcium channel blockers do not cause sodium retention, an undesired effect that is observed with vasodilators including α-blockers, hydralazine, and minoxidil. By contrast, CCBs increase sodium excretion when administered acutely to hypertensive humans and animals (Kazda and Knorr, 1990). Mechanisms involved in the natriuresis evoked by CCBs are associated with various processes such as changes in renal hemodynamic, direct effects on renal tubules or indirect through regulation of vasoactive substances. Several lines of evidence suggest that they possess multiple actions that could be independent of calcium channel blockade. CCBs increase nitric oxide (NO) production both *in vitro* and *in vivo* (Krenek et al., 2001). It is known that NO induces natriuresis by inhibiting sodium reabsorption by the nephron. Another possible mechanism is blockade of T-type channels involved in the renal function. Nifedipine inhibits L-type calcium channels at concentrations much lower than T-type channels, but some other CCBs such as efonidipine block at similar concentration T-type and L-type calcium channels. T-type calcium channels have been identified in vascular smooth muscle cells that are involved in renal blood flow (Godfraind, 1994). In renal tissue, L-type calcium channels are found only in the afferent arterioles, while N-type and T-type calcium channels are located in both efferent and afferent arterioles. Therefore, CCBs that block either T-type or N-type calcium channels may exert renoprotective effects through dilatation of the efferent artery; this avoids hyperfiltration injury of the glomerulus. It has been established that T-type CCBs exert a renal protective action by ameliorating glomerular microcirculation via vasodilator activity on both afferent and efferent arterioles. Additionally, blockade of the T-type Ca channel suppresses inflammatory processes, renin-angiotensin-aldosterone system activation, and oxidative stress. Honda et al. (2001) have compared compounds with different actions on renal afferent and efferent arterioles and have examined whether these CCBs exert divergent actions on natriuresis. Their effect on renal arterioles obviously depends on the molecule so far examined. Intravenous infusion of nifedipine (L-type blocker), efonidipine (L/T-type blocker), or mibefradil (predominant T-type blocker) into anesthetized dogs elicits similar, albeit modest, reductions in blood pressure. The CCB-dependent urinary nitrate/nitrite excretion is dose-dependent but it shows no differences between the various CCBs, indicating that they have similar action on NO production and that this action could not account for the differences observed between drugs in their renal hemodynamic and natriuretic actions. It is proposed that the inhibition of tubular sodium reabsorption associated with the increased post-glomerular blood flow are involved in the natriuretic action of CCBs.

Thus in various situations, hemodynamic studies point to major differences between CCBs, not only in potency but also in selectivity of action. This emphasizes the observation that even drugs having a similar specific molecular target may behave differently *in vivo*, because of the uneven distribution of this target among organs, which become important sites of action in some pathophysiological situations.

## Key information about clinical usefulness of CCBs

### The therapeutic indications of CCBs

The most common indications of CCBs are hypertension and other major cardiovascular disorders. Because vascular risk factors such as hypertension, obesity, and diabetes have been considered as potentially modifiable risk factors for Alzheimer disease, as well as vascular dementia (Barnes and Yaffe, 2011), management of cognitive disorders are included under the same list of indications. A second list comprises neurological disturbances.

In the United States, eight of the major CCBs are currently marketed. Their indications and adverse effects depending on the specific drug are summarized on Table 2.

**Table 2:** **Calcium Channel Blockers currently marketed in the United States**.

**Drug**	**Proprietary name**	**Indications, USA**
Amlodipine	Norvasc	Hypertension; Chronic, stable, and vasospastic angina
Diltiazem	Tiazac; Cardizem; Cartia; Dilacor	Hypertension; chronic, stable, and vasospastic angina; atrial fibrillation or flutter; paroxysmal supraventricular tachycardia
Felodipine	Plendil	Hypertension
Isradipine	Dynacirc	Hypertension
Nicardipine	Cardene	Hypertension; angina
Nifedipine	Adalat; Procardia	Hypertension; angina
Nisoldipine	Sular	Hypertension
Verapamil	Calan; Covera; Verelan	Hypertension
		Angina
		Atrial fibrillation
		Or flutter
		Paroxysmal supraventricular
		Tachycardia

In addition, the dihydropyridine Clevidipine an ultra-short acting drug is approved by the FDA for perioperative use as injectable emulsion in case of severe hypertension in cardiac or non-cardiac intervention. The half-live is of about 2 min due to esterase hydrolysis. The drug is marketed under the name Cleviprex. In Belgium, in addition to verapamil and diltiazem, 11 dihydropyridines are marketed, including those listed in Table 2: Barnidipine (Vasexten), Lacidipine (Motens), Lercanidipine (Lercanimylan, Zanidip), Nimodipine (Nimotop), Nitredipine (Baypress).

Treatments accepted globally with CCBs comprise stable angina and include supraventricular arrhythmias treated with non-dihydropyridine CCBs, but exclude systolic dysfunction. As far as management of hypertension is concerned, the medical community at large is reaching a consensus based on evidence in recommending CCBs in initial treatment of hypertension as reported in major guidelines such as JNC 8 (James et al., 2014) and NICE Clinical Guidelines 127 (http://www.nice.org.uk/CG127) that approves combination with a diuretic in patients with diabetes. An algorithm support of treatment is provided but isn't imposed on physician best clinical judgment for adult patients and for black people of any age (Go et al., 2014).

Meta-analysis comparing clinical effectiveness within dihydropyridine-type CCBs did not indicate major differences (McDonagh et al., 2005). However, based on home BP monitoring, a crossover study of amlodipine vs. nifedipine showed that amlodipine had a lower antihypertensive effect with a lesser pulse rate during the critical morning period (Ryuzaki et al., 2007). More head-to-head studies are needed since pharmacological differences have been observed in the profile of the various dihydropyridines as reported in this paper from experimental observations. This is important when considering validity of translating pharmacological data to patient's treatment. There is a current tendency for the prescription of combinations of antihypertensive drugs. This question has been discussed in another recent publication (Godfraind, 2014).

### The controversy on the safety of CCBs

In the early 1990s questions on the safety of CCBs raised when lidoflazine that had been considered as a very promising therapeutic agent (Jenkins et al., 1981) was withdrawn after publication that its effects were both beneficial and detrimental. The main observation reported is summarized in the following sentences: “During the randomized, placebo-controlled phase of the study with 7-week treatment periods, 9 of 11 patients who completed this phase of the study preferred lidoflazine and all demonstrated improved exercise capacity with lidoflazine compared to placebo. However, three patients developed malignant ventricular arrhythmias, and 1 died while taking lidoflazine, resulting in termination of the study” (Cannon et al., 1990). Arrhythmias have been associated with QT prolongation (Ridley et al., 2004). Therefore, a major question was whether or not such observations were related to class effects or just to a specific chemical structure. Analysis of the literature on CCBs was a rational approach to this question. Meta-analysis of clinical trials of nifedipine was published in 1995 (Furberg et al., 1995). Conclusion of this publication was that moderate to high doses of the short-acting nifedipine increased mortality in patients with coronary artery disease (CHD). This conclusion was refuted by other authors (Opie and Messerli, 1995; Opie et al., 2000; Opie and Schall, 2002) who didn't nevertheless recommend fast administration of nifedipine because of the occurrence of the cardiovascular reflex to acute hypotension. The controversy ended after the publication of ALLHAT a large antihypertensive trial sponsored by the National Heart, Lung, and Blood Institute (Group TAOaCftACR, 2002; Chrysant, 2003). In more than 30,000 high-risk patients with hypertension, this trial compared amlodipine (CCB), lisinopril (ACEI), and chlorthalidone (diuretic), respectively on CHD. No differences occurred in primary end point (combination of fatal CHD and acute myocardial infarction). A large series of analytical and commentary papers followed the publication, highlighting the importance of these findings for the management of patients with hypertension (Leenen et al., 2006). ACTION trial, which studied clinical outcomes in 7,665 patients of 63.5 years mean age, extended to nifedipine GITS the safety conclusions obtained from ALLHAT (Poole-Wilson et al., 2004). Nowadays the controversy on safety of CCBs is closed.

### The action of calcium channel blockers in cardiac ischemia

#### Early clinical studies of CCBs in ischemic heart disease

Ischemic Heart Disease affects the supply of blood to the heart. Blood vessels might be blocked due to deposition of cholesterol in their walls. This reduces the supply of oxygen and nutrients to the heart muscles. This may eventually lead to destruction of an area of heart tissue, inducing a heart attack. Ischemic heart disease is the most common cause of death in many countries around the world. Causal factors including hypertension have been listed above. The clinical aspects of ischemic heart disease are usually expressed by Angina Pectoris, an acute chest pain attributed to chronic stable effort angina, to vasospastic angina, to unstable angina, and acute myocardial infarction. Heart failure might follow a resulting weakness of the heart muscle.

#### Chronic stable effort angina

In the 1970s, about one century after the introduction of nitroglycerine by William Murrel (1853–1912) in the management of angina pectoris (Smith and Hart, 1971), it was reported that agents other than nitrates treat efficiently this pathology. β-Blockers have preceded CCBs, therefore several trials have been designed comparing CCBs with propranolol as well as over placebo.

##### The action of nifedipine in stable effort angina

Nifedipine action has been widely investigated in patients suffering of stable angina. Here are briefly reported earliest studies that established the therapeutic action of nifedipine in angina pectoris. Its action over placebo appeared to be highly significant (Terry, 1982; Sherman and Liang, 1983). Exercise-induced ST segment depression was reduced after 20 mg nifedipine (sublingual) (Hopf et al., 1983). This effect was also observed after oral administration and it was dose-related (Hopf et al., 1983). This beneficial response was also evoked after intravenous and intracoronary administration. When given orally at 20 mg three times per day, nifedipine action persisted during prolonged studies. When nifedipine and nitroglycerin were given sublingually, the authors have estimated the following parameters: work load, maximum heart rate, blood pressure at rest while seated on bicycle, maximum systolic blood pressure measured by indirect measurement using a mercury column manometer, maximum rate pressure product, ST-segment depression at controlled heart rate. Results showed clearly that nifedipine increased total work and decreased the depression of the ST-segment. Nifedipine was also compared to β-blockers (Lynch et al., 1980; Dargie et al., 1981). Effects of nifedipine (60–90 mg per day) monotherapy and propranolol (240 mg per day) monotherapy on symptoms, angina threshold, and cardiac function in patients with chronic stable angina were studied in a placebo-controlled double-blind crossover study. After a 2-week placebo period, patients were randomly ascribed to receive either nifedipine or propranolol for a 5-week treatment period, after which they crossed over to the alternative regimen. All 21 patients were men with chronic stable angina pectoris, 13 of whom had symptoms both at rest and on exertion. In patients taking either nifedipine or propranolol, New York Heart Association functional class improved and nitroglycerin consumption decreased. Nifedipine and propranolol were equally effective in relieving exertion ischemia. Exercise wall motion also improved with both drugs. Propranolol treatment decreased exercise cardiac output by 14 percent (*p* = 0.01) through its effect on heart rate. Nifedipine had the advantage of preserving cardiac output during exercise (Higginbotham et al., 1989). The antianginal effects of nifedipine and propranolol (alone or in combination) compared with placebo were examined in a double-blind clinical trial that included 16 patients with chronic stable angina triggered by effort. Each of the active drugs significantly reduced frequency of chest pain and nitroglycerin consumption. The combination of nifedipine and propranolol increased significantly the effectiveness. About 60 percent of all episodes of ST segment depression were painless and responded to therapy as did episodes associated with chest pain (Dargie et al., 1981). Studies realized in the 1990s and later reinforced evidence of the anti-ischemic effect of β-blockers and CCBs by showing that dihydropyridine-CCBs and β-adrenergic blocking agents are similarly effective in effort agina associated with hypertension (Pfisterer et al., 2010). Opie (2000) noted that safety problems occurred with β-blockers in a study with 12 550 hypertensive patients, those taking β-blockers had a 28% higher risk in developing diabetes whereas this was not observed in those treated with CCBs. He pointed out that the therapeutic option could depend both on the heart and on the patient. When treating an active middle-aged man, preservation of the quality of life must involved exercise training and sexual function. Therefore, there are good arguments for prescribing a CCB. An algorithm was produced for therapy of patients with stable ischemic heart disease (SIHD). A list of appropriate recommendations is available in the document (Fihn et al., 2012). When looking for a better control of heart rate, it is a reasonable combining β-blocker and CCB for angina. The Anglo Scandinavian Cardiac Outcome Trial (ASCOT) (Dahlof et al., 2005) and European Lacidipine Study on Atherosclerosis (ELSA) (Zanchetti et al., 2002) trial compared a β-blocker-based regimen to a CCB-based treatment. In ELSA, for a similar reduction in BP a better protection toward atherosclerosis was observed with CCB than with β-blocker. ASCOT was a multicenter, prospective, RCT comparing amlodipine to atenolol in 19,257 patients aged 40–79 years with hypertension and at least three other CV risk factors. The amlodipine-based regimen prevented more major CV events and induced less diabetes than the atenolol-based regimen. Thus, clinical studies favored CCBs for the management of hypertensive patients with SIDH.

##### The action of verapamil and diltiazem in stable effort angina

Verapamil was initially considered as a β-blocker (Kaltenbach and Zimmerman, 1968; Nayler et al., 1968), but was later characterized as a calcium antagonist by Fleckenstein et al. (1969). Several controlled clinical trials have established the efficacy of verapamil over placebo (Opie, 1989). Most of the controlled clinical trials (CRTs) dealt with comparison of verapamil vs. propranolol, using as an objective criteria as change in ST segment during and after exercise (Hopf et al., 1983). The usual procedure was to estimate the duration of a given work on the appearance of conventional electrocardiographic positivity (1 mm ST depression). As reported by de Ponti and Vincenzi (1981), the duration of work, expressed in second, increased significantly after verapamil administration and this effect was dose-dependent. Furthermore, after intravenous injection, the effective dose of verapamil was much lower than the dose required per oral route, an indication that the bioavailability of verapamil is reduced per os. In pharmacokinetic studies, the bioavailability of verapamil in human was estimated of 22% (Eichelbaum and Somogyi, 1983). This estimate was confirmed in functional studies, when the reduction of ST depression for a given work load was measured in patients treated with various doses of verapamil (Hopf et al., 1983).

Like nifedipine, it was compared with β-blockers by measuring the reduction of pain attacks or of nitroglycerin consumption. It appeared that verapamil 360 mg/day was equiactive to propranolol 300 mg/day (Sandler et al., 1968; Livesley et al., 1973; Johnson et al., 1981; Leon et al., 1981; Frishman et al., 1982; Sadick et al., 1982; Tan et al., 1982; Findlay et al., 1987). Since there is a dose-dependency for both agents, it is obvious that the superiority of one agent against the other cannot be estimated by comparing a single dose of each drug. When other criteria were used, it appeared that if β-blockers delayed ECG alterations more than angina, verapamil, and nifedipine delayed angina more than ECG alterations, indicating the existence of a qualitative difference between β-blockers and CCBs (de Ponti and Vincenzi, 1981).

Diltiazem has also been studied in stable angina and compared to placebo and propranolol. Effects observed were similar to verapamil. Bradycardia was observed like with verapamil, likely related to blockade of Ca_v_3 channels (Striessnig, 2001).

Usually, those comparisons used diltiazem 9 mg four times daily, propranolol 60 mg four times daily against placebo. According to Schroeder et al. (1985), diltiazem decreased the sub-maximal and maximal degree of exercise-induced ST segment depression by over 50% compared to placebo (*P* < 0.01 vs. placebo). Diltiazem resulted in a higher exercise left ventricular ejection fraction compared to placebo, propranolol or the combination of diltiazem and propranolol (all less than *P* < 0.05). The sinus bradycardia occurred in patients required dose reduction.

*In addition to chronic stable effort angina*, the clinical use of CCBs for treatment of the other clinical types of ischemic heart disease including silent angina, vasospastic angina, unstable angina, and acute myocardial infarction are reported in recent Guidelines.

*Side effects* of verapamil, nifedipine, and diltiazem observed during studies reported above.

Drug-induced cardiac ischemia results from a sympathetic reflex in response to a rapid lowering of blood pressure, which may provoke cardiac ischemia due to too fast IV administration. This has prompted a revision of the mode of administration of nifedipine, which is nowadays given by oral route in slow releasing (GITS) preparation. Earliest observations were the basis for the short-term controversy on the safety of CCBs discussed above. The more frequent side effects of nifedipine, verapamil, and diltiazem were related to their action on smooth muscles; with verapamil, about 70% of the patients complained of constipation; with nifedipine, the major side-effect was ankle edema, a side-effect also reported with diltiazem. Due to their potent negative inotropic effect, overdosage of verapamil and diltiazem have been avoided in order not to aggravate symptoms of cardiac insufficiency.

### Calcium channel blockers in cardiac arrhythmias

In slow response tissues such as sinoatrial and atrioventricular nodes, non-dihydropyridine CCBs (nd-CCBs) do block Ca current that generate slowly propagating action potentials, this action displays antiarrhythmic effects. Acute myocardial infarction may convert fast conducting tissue such as ventricular myocardium and Purkinje fibers into slow response tissue. In ischemic areas, ionic changes cause partial depolarization in resting cells supporting slow Ca currents and leading to conduction blocks. Such blocks play a crucial role in the development of reentrant pathways. These processes are involved in the incidence of premature beats and ventricular tachycardia. In 1971 verapamil that was known to inhibit arrhythmia induced by ouabain, was used to reduce the ventricular rate in atrial fibrillation (Schamroth, 1971). It has been later ranged in class IV antiarrhythmics (Singh, 2000). Its action is due to interaction with intracellular binding sites different from the dihydropyridine receptor. Verapamil and diltiazem *in vivo* are powerful antiarrhythmic agents. Dihydropyridines usually evoke reflex tachycardia resulting from increase in sympathetic tone, masking their slight negative chronotropic effect. Verapamil and diltiazem are recommended in supraventricular tachycardia (Page et al., 2016). Intravenous verapamil produces the conversion of reentrant supraventricular tachycardia, diltiazem being less effective (Schamroth and Antman, 1983) and (Camm et al., 2010). Intravenous injection of verapamil and diltiazem in atrial fibrillation results in reduction of the ventricular response (Nademanee and Singh, 1988) an effect confirmed in the Verapamil plus antiarrhythmic drugs trial (De Simone et al., 2003). However, in the presence of anomalous bundle, verapamil, and diltiazem are contraindicated and the ESC guidelines recommend a treatment by catheter ablation for the management of atrial fibrillation (Camm et al., 2010).

### Calcium channel blockers in hypertension and HT complications

According to 2016 reports of WHO and of Medscape, globally, the overall prevalence of raised blood pressure in adults aged 25 and over was around 40% in 2008. The number of hypertensive adults worldwide was estimated to 1.1 billion in 2015 with a disparity among countries, the prevalence being lowest in wealthy countries. This could be explained by diet and drug treatment control. In view of disability or death due to complications, such as cardiac ischemia, kidney insufficiency, stroke and dementia, it is mandatory to evaluate the efficacy of medications.

**Figure 6:**
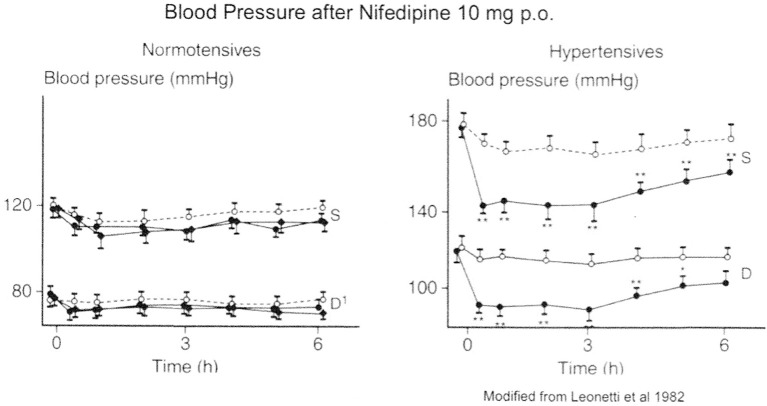
**Action of nifedipine on Blood Pressure: difference in normotensive and hypertensive patients**. Modified from Leonetti et al. (1982).

On the basis of their potent vasodilator properties, CCBs have been proposed as antihypertensive drugs, however CCBs may hardly be classified among vasodilators for their therapeutic action. Indeed, *in vivo*, they mainly act on the arterial bed and don't modify venous tone. As a consequence they don't evoke orthostatic hypotension. They reduce vascular resistance (and afterload) inducing a diminution of blood pressure (BP). At nifedipine therapeutic dosage, BP reduction is observed in patients with hypertension and not in normotensive individuals as shown on Figure 6. Further studies confirmed this selective BP effect with other CCBs including verapamil, nitrendipine, diltiazem, tiapamil, and isradipine but not with propranolol or captopril (Bühler et al., 1985). On the blood pressure of hypertensive SHR and normotensive WKY Knorr and Garthoff (1984) have compared the activity of nitrendipine and hydralazine. They found that the vasodilator hydralazine was equipotent in both strains, but that nitrendipine evoked a lower reduction of blood pressure in WKY than in SHR. The free cardiovascular tissue concentration of CCBs is similar in SHR and WKY after chronic oral treatment of respectively nisoldipine (80 mg/kg/day) and amlodipine (10 mg/kg/day). However, there is a significant reduction of BP in SHR and no change is observed in WKY (Godfraind et al., 1991; Morel and Godfraind, 1994). Such studies confirmed that considering their CV activity profile, CCBs are differing from classical vasodilators. Morel and Godfraind have shown that CCBs have a higher affinity for specific binding sites in vessels of hypertensive rats than in vessels of normotensive ones. The augmented sensitivity of BP to the effect of CCBs in hypertensive rats when compared to normotensive controls is likely due to this higher affinity. This increased affinity has been related to different levels of resting membrane potential of arteries leading to a different proportion of inactivated calcium channels (Morel and Godfraind, 1994). Furthermore, the CCB-dependent prevention of endothelial dysfunction in SHR vessels facilitates their relaxation resulting from calcium channel blockade (Krenek et al., 2001). CCBs efficacy has also been reported in patients with low renin activity whose hypertension is insensitive to β-blockers. Animal experiments show that the interaction of CCBs with the renin–angiotensin system is complex (Kyselovic et al., 2001). Like ACEI, CCBs exhibit a natriuretic effect. This action occurs without significant alteration in renal plasma flow or in glomerular filtration rate (Zanchetti and Stella, 1988). As shown by Honda et al., the natriuretic effect is related to the structure of the DHP-CCB: efonidipine, which mainly dilates afferent artery is more efficient than nifedipine, which mainly dilates efferent artery (Honda et al., 2001; Hayashi et al., 2007). This difference may be accounted for by interaction with T-type Ca channels (Hayashi et al., 2007), but other actions have been suggested. The natriuretic action supports the use of CCBs in monotherapy of hypertension. It is hidden during prolonged treatment but is manifested by a fall in natriuresis observed after drug withdrawal. CCBs are currently combined with other antihypertensive drugs such as ACEI or ARB for hypertension management (Godfraind, 2014). Long-term administration of CCBs to SHR opposes cardiac pathological remodeling and induces substantial regression of established LV hypertrophy thereby improving cardiac function (Kyselovic et al., 2001). Preserve trial was designed to compare enalapril and nifedipine on regression of LV hypertrophy at equivalent BP reduction (Devereux et al., 2001). Treatment began with enalapril 10 mg or nifedipine GITS 30 mg and matching placebo. If required, enalapril or nifedipine were increased to respectively 20 mg or 60 mg, hydrochlorothiazide (HCTZ; 25 mg) and then atenolol (25 mg) were supplemented if maximum dose did not control BP. More supplemental treatment with HCTZ was required in ACEI-treated patients than in CCB-treated ones. Importantly the study showed that both regimens similarly reduced to the normal range LV mass index and relative wall thickness during 1 year of treatment in about 50% of patients. The initial hypothesis was that normalization of BP and LV systolic load evoked regression or prevention of heart hypertrophy. However, several mechanisms might be involved (Godfraind, 2014). As demonstrated in various clinical trials (Tocci et al., 2015), BP lowering reduces the incidence of stroke and myocardial infarction. Head-to-head comparisons are incomplete but ASCOT trial (Sever et al., 2003) and several meta-analyses indicate that CCBs offer a better protection against stroke and myocardial infarction than angiotensin receptor blockers (Wang et al., 2007). This is consistent with survival to malignant hypertension of CCB-treated stroke-prone SHR (Godfraind and Salomone, 2015).

Alteration of renal function is another complication of hypertension. Diabetic nephropathy is particularly problematic. Verapamil and efonidipine (not marketed in United States) are equally efficient in this pathology (Sasaki et al., 2009). As pointed out above, studies on renal vessels showed that efonidipine dilates the efferent artery (Ozawa et al., 1999). This is attributed to a different action on T-type Ca channels. Verapamil act also at their level but amlodipine and nifedipine are weak blockers of those channels (Perez-Reyes et al., 2009). A role for amlodipine in renal disease is reported in combination therapies with other antihypertensive agents (Godfraind, 2014).

Hypertension impairs cognitive function, a harmful effect well-recognized nowadays (Moskowitz et al., 2010). It is clinically manifest as dementia that is recognized by cognitive decline eventually resulting in vascular dementia or Alzheimer disease globally diagnosed in about 40 million people. The evidence of the relation with hypertension has been hardly accepted because it requires longitudinal study (Staessen and Birkenhager, 2004). It is now believed that midlife hypertension has a deleterious influence on late-life cognitive function (Iadecola et al., 2016). Only few randomized clinical trials were conducted to study influence of antihypertensive therapy on the evolution of established dementia, their protocols were not similar regarding preceding clinical conditions and drugs used for treatment of selected patients. The first study reporting significant reduction of dementia incidence was a vascular dementia project part of the European Working Party on High Blood Pressure in the Elderly (SystEur) designed to investigate whether cardiovascular complications of isolated systolic hypertension in people aged 60 years or over could be reduced by nitrendipine (Staessen et al., 1997). The primary end point was fatal and non-fatal stroke. Because of the demonstration of a significant benefit for stroke, the trial initially planned for 4 years was limited to 2 years. During this period, the incidence of dementia was significantly reduced by 50%, expressed in cases per 1,000 patients-years, it was equal to 7.7 in the placebo group and to 3.7 in the nitrendipine group. The total number of cases was equal to 32. After this period, all subjects were invited to continue the trial for 2 year with the dosage of nitrendipine administered during the first period, which included patients previously treated with placebo. At the end of the period of 4 years, the total number of dementia cases rose to 64. The group treated for 4 years with nitrendipine showed a significant lower number of dementia than the group initially treated by placebo, which had received nitrendipine during only 2 years, indicating a positive effect of the duration of nitrendipine therapy (Forette et al., 2002; Hanon and Forette, 2004). There is not yet confirmation of Syst-Eur study by longitudinal trial. A multicenter trial is in progress with nilvadipine in mild to moderate Alzheimer disease (Lawlor et al., 2015; Meulenbroek et al., 2016). However, there are published cross-sectional studies based on databases consultation. The most extensive is a Taiwan study consisting on analysis of the National Health Insurance Research Database dated from 2000 to 2010. It comprised 82,107 hypertensive patients aged 60 years or more. The annual incidence of dementia was 3.9 cases in the CCBs group vs. 6.9 per 1,000 persons-years in the comparator group (*P* < 0.01). Interestingly those data are not far from those of the longitudinal Syst-Eur study (Wu and Wen, 2016). The two other studies from databases are also confirmatory of the preventive action of CCBs in dementia (Feldman et al., 2016; Hwang et al., 2016). In the sub-study of the PROGRESS (Preventing Strokes by Lowering Blood Pressure in Patients With Cerebral Ischemia) trial, after treatment with ACEI and/or diuretics, it was shown that the risks of dementia and of cognitive decline among recurrent stroke patients were reduced to 34% and 45%, respectively, over mean time of 3.9 years, but those of non-recurrent stroke patients were not significantly affected (Tzourio et al., 2003). In SCOPE (The Study on Cognition and Prognosis in the Elderly), examination of cognitive function state with angiotensin II receptor blockade (ARB) against placebo showed no significant changes (Lithell et al., 2003). The effect of diuretics (perindopril plus indapamide) was examined in the HYVET-COG (Hypertension in the Very Elderly Trial assessing Cognitive decline and dementia incidence) study, the prevalence rate of dementia associated was reduced from the non-users but not significantly different after a mean of 2.2 years since the start of treatment. According to authors, this could be due to insufficiency of population tested (Peters et al., 2008).

It is obvious that further data are required for a robust evidence of the unique protective effect of CCBs treatment in prevention of dementia.

### Calcium channel blockers in atherosclerosis

The increase of plasma lipid levels constitutes risk factor for arteries. Clinical evidence indicates that the progression of the disease can be inhibited by sustained lipid-lowering therapy. As reported by Henry in a review on atherosclerosis (Henry, 1990), lesion formation is depending on calcium-regulated cellular processes such as chemotaxis, adhesion, migration, proliferation, lipid uptake, and necrosis. By acting on cell calcium uptake with calcium chelating agents, lanthanum trichloride and CCBs, atherogenesis in fat-fed animals may be retarded in the absence of hypolipidaemic effects. Fleckenstein and Colleagues confirmed those studies in fat-fed animals (Fleckenstein, 1987; Fleckenstein-Grun et al., 1992). Clinical studies were designed with CCBs in order to examine whether a similar result could be achieved in patients without influencing plasma lipid levels. The first was the International Nifedipine Trial on Antiatherosclerotic Therapy (INTACT) (Lichtlen et al., 1990). This trial showed significantly reduction of the appearance of newly formed coronary lesions in patients exposed to nifedipine. However, existing lesions were not modified. Unfortunately this trial didn't examine blood pressure. The latter information is needed to establish an action of CCB over other antihypertensive agents. Such requirement was included in the Verapamil in Hypertension and Atherosclerosis Study (Rosei et al., 1997; Zanchetti et al., 1998), which compared verapamil (240 mg OD) and chlortalidone (25 mg OD). Dissimilarities in carotid wall changes were small but greater incidence of CV events was noted in patients randomized to chlortalidone than to verapamil (*P* < 0.05). Differences in the incidence were paralleled by small differences in carotid wall changes suggesting that clinical and prognostic significance might depend on small effects on carotid plaques. In INSIGHT study, nifedipine GITS was significantly more efficient on intimal-media thickness than co-amilozide (hydrochlorothiazide + amiloride) (Rosenthal, 2002). Amlodipine effect on the progression of early coronary atherosclerosis was tested in PREVENT (Pitt et al., 2000) by evaluation of the rate of atherosclerosis in the carotid arteries together with monitoring the rates of clinical events. As compared to the placebo group, amlodipine had a significant effect in slowing the 36-month progression of carotid artery atherosclerosis but not in the rates of all-cause mortality or major cardiovascular events. Nevertheless, amlodipine use was associated with fewer cases of unstable angina. Other randomized trials including diuretics have confirmed that CCBs reduced progression of carotid lesions (Borhani et al., 1996; Simon et al., 2001). The ELSA trial (Zanchetti et al., 2002) compared carotid intimal-media thickness (IMT) changes over 4 years in patients receiving either a β-blocker or a dihydropyridine. Lacidipine exerted greater protection on carotid IMT progression and on number of new plaques increase than atenolol, despite a smaller BP reduction. In the analysis of the REGRESS Study Group trial, it appeared that co-administration of CCB, amlodipine or nifedipine with pravasatin caused the largest reduction in the appearance of new angiographic lesions, indicating that drug-combinations act synergistically and confirming the therapeutic action of CCBs in atherosclerosis (Jukema et al., 1996). This therapeutic action has been demonstrated in ApoE-deficient mice exposed to a lipid-rich Western-type diet (WD) (Nakashima et al., 1994), which even on a normal diet (ND) exhibit endothelial dysfunction (Plump et al., 1992; Bonthu et al., 1997; Barton et al., 1998; d'Uscio et al., 2001) proposed to initiate atherogenesis (Libby and Galis, 1995; Libby et al., 1995; Barton and Haudenschild, 2001). The latter is associated with increased peroxidation of plasma lipids, LDL and VLDL, as well as augmented susceptibility of lipoproteins to be oxidized *ex vivo* (Hayek et al., 1994).

**Figure 7:**
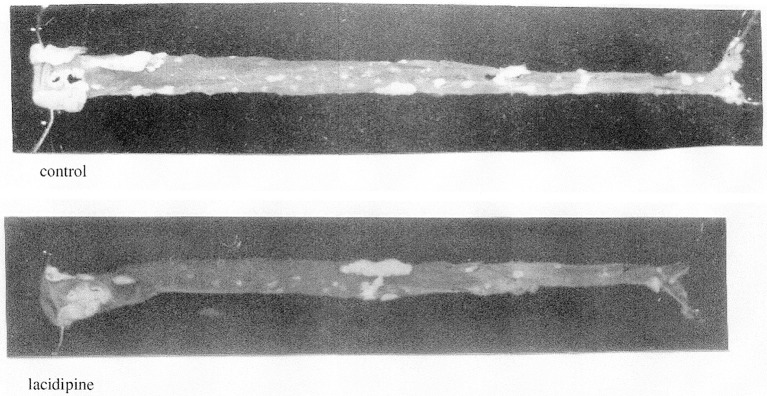
**Atherosclerotic lesions in aorta of apo-E deficient mice treated or not with lacidipine**. Modified from Kyselovic et al. (2005).

In experiments from our group on ApoE KO mice (Kyselovic et al., 2005), lacidipine not only prevented endothelial dysfunction but also the development of atherosclerosis as shown in Figure 7, an action occurring without high cholesterol reduction. Indeed lacidipine treatment suppressed the loss of acetylcholine-evoked relaxation in aorta of mice fed Western lipid-rich diet. It abolished increase in kidney TBARS markers of oxidative stress. The CCB treatment also normalized ET-1 plasma level and elevated PRA. A marked nephroprotective effect was related to decrease of oxidative stress and improvement of renal blood flow. Some effects reported in ApoE-deficient mice had also been observed in rabbit fed atherogenic diet (Becker et al., 1991).

### Beyond the cardiovascular system

Blockade of plasma membrane Ca entry through VOCs allowed therapeutic actions in neurological disturbances such as epilepsy, migraine, pain particularly neuropathic pain, and sub-arachnoids hemorrhage. When considering neuropharmacological indications, a review of 1986 (Godfraind et al., 1986a), mentioned flunarizine and nimodipine for treating common and classical migraine. Target of this action (cerebral arteries or neurones) remained contentious. Clinical trials examined the protecting action of CCBs from ischemic brain damage. In patients with aneurysmal subarachnoid hemorrhage, nimodipine secured patients at risk. Aged patients benefitted from cinnarizine and flunarizine for the treatment of vertigo and of sleep disorders. Some authors suggested a possible action in a number of forms of epilepsia. An interaction of neuroleptics with calcium channels has been clearly demonstrated (Santi et al., 2002). Therefore, calcium channels might be targets for therapeutic actions beyond the cardiovascular system.

There are experimental trends to extending the action of CCBs in cancer therapy by considering the major function of T-type currents in activating cancer cells (Heady et al., 2001; Panner and Wurster, 2006).

#### Migraine (with a note on vertigo)

As far as migraine treatment is concerned, there is a large body of information confirming the efficacy of flunarizine and nimodipine for the prevention of the migraine attacks, which are reduced in frequency in patients treated with these drugs (Formisano et al., 1991; Luo et al., 2012). Only a few studies include an additional follow-up after discontinuation of migraine prophylaxis with either drug. Nutti et al. reported a single blind evaluation of the efficacy and tolerance of flunarizine (25 patients) in comparison with nimodipine (25 patients) after discontinuation of a 6-month treatment (Nuti et al., 1996). It has also been reported that flunarizine reduced cortical spreading depression (Dora et al., 2003; Ayata et al., 2006), indicating activity on disseminated nervous disorders. Visual aura is the result of cortical spreading depression (CSD) that extends slowly across the cerebral cortex as a wave of neuronal depolarization (Bowyer et al., 2001; Hadjikhani et al., 2001). Headache is a consequence of neurogenic inflammation and activation of trigeminal nucleus caudalis and brainstem nuclei involved in the perception of pain (Moskowitz and Macfarlane, 1993; Waeber and Moskowitz, 2005). In the rat cortex CSD stimulates trigeminovascular afferents and evokes events consistent with the development of headache (Bolay et al., 2002). Thus, CSD is a critical event in the pathogenesis of migraine with aura. Some authors have proposed that nitrergic nerves are involved in this process (Olesen and Jansen-Olesen, 2000; Toda and Okamura, 2003). Mutations in the gene encoding the pore-forming α_1_-subunit of Ca_V_2.1 (voltage-gated P-Q-type) channels have been reported in familial hemiplegic migraine (FHM) (Tottene et al., 2002). They expressed FHM mutants in Ca_V_2.1-deficient neurons. Therefore, a role for Ca_V_2.1 channels could be dominant in the pathogenesis of migraine aura, indicating a potential molecular target for unspecific CCBs such as flunarizine. Alternatively, Ca_V_1 channels (L-type) may also be involved, since they are upregulated in mouse brain subjected to episodes of CSD (Choudhuri et al., 2002). There are other targets, which could account for an action of CCBs (Reuter et al., 2002). It appears that the precise mechanism of action of CCBs in migraine remains conjectural, but identified targets support the clinical observations of their therapeutic benefit (Pietrobon and Moskowitz, 2013).

Vertigo associated with migraine may also be reduced by CCBs (Hain and Uddin, 2003). Cinnarizine and nimodipine are efficacious in otological vertigo (Pianese et al., 2002). This is also true for the action of flunarizine in vestibular neuritis (Corvera et al., 2002). It might be that such therapeutics effects are related to a vascular action (Moskowitz and Macfarlane, 1993).

#### Neuropathic pain

Pain is typically a sign of tissue injury and is usually temporary in duration. With healing, the pain associated with the wound will resolve. Its major role is to warn the individual from further injury. However, in some individuals, this painful experience can result in chronic pain that persists for months or even years after the initial insult. Abnormality of the peripheral, central, and sympathetic nervous system can result in a painful state termed neuropathic. Hence, neuropathic pain represents a chronic pain syndrome with a diverse etiology and perhaps an anatomical cause. L-, N-, and P/Q-types Ca^2+^ channel types are operating in the spinal cord and studies based on the spinal delivery of specific antagonists to high-threshold calcium channels reveal that their blockade, particularly of N-type channels, can prevent or attenuate subjective pain as well as primary and/or secondary hyperalgesia and allodynia. Selective block of N-type channels via intrathecal administration of ϖ-conotoxin GVIA or ϖ-conotoxin MVIIA significantly depresses pain, hyperalgesia, and allodynia in various animal models subjected to experimental situations (Vanegas and Schaible, 2000).

Ziconotide, a synthetic form of the *Conus magus* peptide toxin is a selective antagonist of the N-type Ca^2+^ channel. In animal models, it is antinociceptive of persistent, post-operative and neuropathic pain. It is more potent and specific than morphine on intrathecal administration and doesn't exhibit tolerance, acting on Ca^2+^channels found in high concentration on the superficial laminae of the spinal cord dorsal horn. Pretreatment prevents allodynia and hyperalgesia (Penn and Paice, 2000; Ridgeway et al., 2000; Wang et al., 2000; Smith et al., 2002; Pope and Deer, 2015; Manda et al., 2016).

Gabapentin, 1-(aminomethyl) cyclohexaneacetic acid, is efficacious in epilepsy therapy through interaction with the alpha 2-delta subunit of L-type calcium channel (Striano and Striano, 2008). The gabapentin binding site has been purified from pig brain and gabapentin was identified as the first ligand to interact with the α_2_δ subunit of high-threshold Ca^2+^ channels (Dolphin, 2013). It is preventing hyperalgesia in a number of different models of neuropathic pain through its action at the post-synaptic dorsal horn and is effective in the treatment of neuropathic pain in diabetic neuropathy and post-herpetic neuralgia (Rose and Kam, 2002; Moore et al., 2014). Following sciatic nerve chronic injury, [^3^H]^+^gabapentin binding sites are upregulated in the dorsal horn. The association of α_2_δ with the pore-forming α_1_ subunit of the Ca^2+^ channel modulates channel activity. This indicates that gabapentin could affect Ca channel function indirectly, thereby modifying neuronal excitability (Snutch et al., 2001). The exact mode of action of gabapentin still needs to be better evaluated. No doubt that this investigation shall improve our knowledge about interaction of drugs alleviating pain with calcium channels.

#### Subarachnoid hemorrhage

Brain ischemia, which may be related to vasospasm, is a frequent cause of poor outcome in patients with subarachnoid hemorrhage (Kasuya et al., 2002; Lefranc et al., 2002), Experimental studies have indicated that nimodipine can prevent or reverse vasospasm in the nervous system (Hall and Wolf, 1986). In ischemic tissues, rundown of ionic gradients results in membrane depolarization allowing excessive Ca^2+^ influx that provokes a wide array of metabolic changes resulting in cell death. With reperfusion, extracellular Ca^2+^ increases, and rapid Ca^2+^ overload damages further cells (Siesjo, 1988a,b; Katsura et al., 2000). The most effective way to improve cell survival is blockade of the deleterious Ca^2+^ accumulation by CCBs, which promote the relaxation of cerebral vascular smooth muscle and thereby restore the cerebral blood flow (Ott et al., 2014; Hockel et al., 2016). The neuroprotective effect of CCBs has been documented after both global and focal ischemia in animal experiments (Bentahila et al., 1991; Alps, 1992; Oka et al., 1999, 2000). Studies with cells in culture have shown the ability of nervous cells to adapt to and recover from insult (Katsura et al., 1993). Neuronal and vascular smooth muscle cells are differently regulated by a chronic depolarization but are protected from the deleterious effects by nifedipine and nimodipine (Feron and Godfraind, 1995). Nimodipine is recommended in AHA guidelines for treating vasospasm following Subarachnoid Hemorrhage (Connolly et al., 2012).

## Conclusions

In the various sections of this paper dedicated to history, facts reported by a large number of authors have been put forward. They do enlighten the global cooperative activity of the basic and clinical biomedical community to analysis of the potential of CCBs for treating a large spectrum of diseases from angina pectoris to various forms of dementia. There are some experimental discoveries that haven't been accounted for in this Review. They have been covered elsewhere under the topic “long-term effects” (Godfraind, 2000, 2004), which comprises antioxidant effects (Godfraind, 2005; Godfraind and Salomone, 2015), vascular remodeling actions (Arribas et al., 1999), gene expression and function of major autacoids including angiotensin (Kyselovic et al., 2001), NO (Krenek et al., 2001), endothelin (Godfraind, 2000). It is hypothesized that such long-term effects are involved in the therapeutic action of CCBs, an action that could not only been due to reduction of vascular tone controlling the level of blood pressure but also to BP independent actions. Authors of large RCTs reported above favor this assumption. On the basis of electrophysiological experimentation (Perez-Reyes et al., 2009), the hypothesis cannot be ruled out that in addition to blockade of Ca_v_1.2, blockade of other voltage-operated channels could be of importance for therapy. However, as well as for long-term effects, robust demonstration needs to be supported by clinical data. Much hope is provided by ongoing translational research (Griendling et al., 2016) and by Big Data analysis in the future of Medicines development.

## Author contributions

The author confirms being the sole contributor of this work and approved it for publication.

### Conflict of interest statement

The author declares that the research was conducted in the absence of any commercial or financial relationships that could be construed as a potential conflict of interest.
